# Clinical Effects of the Extract of the Seeds of the Indian Celery—*Apium graveolens*—In Horses Affected by Chronic Osteoarthritis

**DOI:** 10.3390/ani9080585

**Published:** 2019-08-20

**Authors:** Beatrice Battaglia, Mario Angelone, Elena Vera, Giuseppina Basini, Simona Bussolati, Massimiliano Paci, Maurizio Del Bue, Raffaella Aldigeri, Stefano Grolli, Fausto Quintavalla, Roberto Ramoni

**Affiliations:** 1 Dipartimento Scienze Medico Veterinarie, Università di Parma, Via del Taglio 10, 43126 Parma, Italy; 2 Dipartimento di Medicina e Chirurgia, Università di Parma, Via Gramsci 14, 43126 Parma, Italy

**Keywords:** apium graveolens, osteoarthritis, NSAID, natural extract, phytotherapy, chronic orthopedic disorders

## Abstract

**Simple Summary:**

In recent years, the sensitivity and interest of veterinary practitioners and owners both for elderly pets and for those suffering from chronic or degenerative diseases, has increased considerably. As for the horse, both the progression of age, or an intense sport activity, are accompanied by the onset of chronic arthritic forms that affect the animal welfare and significantly limit its movements. To date, the available drugs cannot eradicate the causes of these diseases, whose symptoms are usually treated with anti-inflammatory drugs. These drugs, although extremely effective, due to the side effects, can only be used for short periods of time. As an alternative to these treatments, we investigated here the use of a natural product, the extract of Indian celery seeds, that in humans is employed as a supplement with anti-inflammatory properties, for long lasting treatments of chronic osteo-arthropathies. The improvements obtained and the lack of side effects for prolonged treatments, suggest that also in the equine species the Indian celery seed extract can be successfully administered to ameliorate both well-being and quality of life of subjects suffering from chronic joint diseases.

**Abstract:**

The extract of the seeds from Indian celery, *Apium greaveolens* (CSE), tested in experimental animals (rodents), and in humans affected by chronic osteoarthritic diseases, exhibits anti-inflammatory effects that can be compared, to some degree, to those of non-steroid anti-inflammatory drugs (NSAID). In view of a potential use of CSE in the equine species, it was tested on horses affected by chronic articular pathologies. The trial was performed on 20 horses divided into three different groups, orally treated with 0 (controls), 7.0 or 30 g of CSE BID. Basic orthopedic examinations were conducted, vital signs were observed, and blood samples collected. Improvement was observed at the highest dosage tested (30 g of CSE BID), as reflected in the score values of three clinical parameters, (i) amplitude and (ii) sensitivity to passive flexion and (iii) flexion test. Since the improvement of these parameters can be correlated with a lower perception of the pain, the present data suggest that the CSE treatment can have an analgesic effect in horses affected by chronic osteoarthritic diseases.

## 1. Introduction

Chronic osteoarticular pathologies, which affect about 60% of athlete and elderly horses [1], are the most disabling diseases in the equine species. Osteoarthritis (OA) in horses is characterized by a slow course, due to the progressive loss of functionality caused by cartilage wear, bone remodeling and changes in the surrounding soft tissues [2]. The pain and the limitation of the movements of the joint that derive from OA are the factors that determine the inability of the subject to work, and, therefore, the main aspects to consider to set up a correct therapy are aimed at providing a good quality of life to the animal [3,4]. In the clinical practice, the ideal treatment for OA should determine the reduction of both pain and clinical signs (symptomatic therapy), and stimulate, at the same time, regenerative processes that bring the joint to a renewed structural and functional condition [5]. The therapeutic management of OA is generally based on the use of non-steroidal anti-inflammatory drugs (NSAIDs) or steroids that often cause significant undesirable effects. For this reason, in recent years, practitioners often opt for multimodal therapeutic protocols that introduce the use of non-pharmacological treatments (laser therapy, shock waves, acupuncture, tecar, etc.) and nutraceuticals [6,7,8,9]. As a contribution to this novel approach, the present study evaluates the efficacy and tolerability of a natural extract of seeds from Indian celery, *Apium graveolens*, (CSE) [10] in horses suffering from OA. This extract, which is already employed as an alternative, or in combination to NSAIDs for the treatment of chronic joint diseases in humans, is classified in the group of safe substances by the United States FDA (Title 21, Code of Federal Regulations 182.10 and 182.20) and is registered in Australia in the TGA (Australian Registrar of Therapeutic Goods—Nr: L55831). CSE, in addition to volatile oils, such as limonene, phthalide (2–3% *V*/*W*), and flavonoids (apigenin and apiin), contains several components (Sedanolide, Senkyunolide-N, Senkyunolide-J, 3-hydrossimethyl-6-methoxy-2,3-dihydro-1H-indol-2-ol, Acetylated product of compound 4, l-tryptophan, 7-[3-(3,4-dihydroxy-4-hydroxymethyl-tetrahydro-furan-2-yloxy)-4,5-dihydroxy-6-hydroxymethyl-tetrahydro-pyran-2-yloxy]-5-hydroxy-2-(4-hydroxy-3-methoxy-phenyl)-chromen-4-one) that inhibit cyclooxygenase (COX) [10]. Therefore, CSE composition allows the hypothesis that it can act as an inhibitor of inflammation [11,12,13]. Interestingly, although CSE exerts its anti-inflammatory effects with mechanisms similar to those of NSAIDs, it doesn’t cause the unwanted side effects (in particular lesions of the gastrointestinal mucosa) that are determined by prolonged treatments with these drugs. The reason probably resides in a gastroprotective effect of some of the CSE components, as described in rodents treated in combination with NSAIDs, that is probably mediated through non-prostaglandin mechanisms [14]. As reported in the sheet accompanying the commercial formulation, in humans CSE is administered orally at a dosage of 1.04 g BID (corresponding to 16 mg/kg/day for a subject weighing 65 kg). Then, after two weeks, it is reduced to one half for the duration of the treatment. The amelioration of symptoms, that had appeared after two weeks, is maintained as long as the CSE is administered.

## 2. Materials and Methods

### 2.1. CSE Powder and Dosages

The CSE powder used in this study has been provided by Synergia Life Sciences Pvt. Ltd., Mumbai, India. The powder was obtained by the fluid extraction of the seeds of Indian celery according to a procedure protected by a patent (Patent no US6,576,274 B2, 10 June 2003, Butters et al. United states patent), which allows the obtention of about 76 mg of CSE powder, starting from 1.0 g of celery seeds. Since CSE, in agreement with the European regulations, is not considered a drug, but rather a simple food product (Reg. CE 183/2005, Reg. CE 767/2009 and Reg. CE 68/2013), for the evaluation of its biological activity in-vivo, it has not been necessary to follow the protocols prescribed in the case of clinical trials with pharmaceuticals in live animals. This study was submitted to the Committee for Animal Ethics of the University of Parma (approval number 12/CE/2019), and the experiments were conducted in accordance with the approved guidelines. In the present investigation the CSE was tested in the horse at two different dosages, i.e., 7.0 g BID and 30 g BID, corresponding to 23.3 and 100 mg/kg for a subject weighing 600 kg, respectively.

7.0 g is the minimum amount of CSE powder that can be measured with sufficient accuracy and repeatability in the field and is two times higher than the mean value of the dosages adopted in the two phases of the CSE treatment in humans. The high 30 g BID value of the second dosage was chosen to evaluate both different responses of the equine species to the CSE treatment compared to humans, and the eventual side effects of the CSE. To facilitate the administration of the CSE by the stablemen, differently from the human species, the dosages were kept constant for the entire duration of the treatment.

### 2.2. Horses

Twenty horses of different races, age and sex, and attitude (Table S1: Characteristics of the subjects enrolled in the trial and experimental data collected), all affected by chronic OA were enrolled in five equestrian centers near Parma (Italy). While for some of them both OA symptoms and pain behavior were clear, for others, they could be detected by the practitioners during the clinical visits (see below). The horses were all subjected to the same feeding regimen and housing. They were randomly divided into three groups according to the dose of CSE to be administered (Table S1: Horses and treatments)—0 g (control group, 12 horses), 7.0 g BID (group A, 5 horses) and 30 g BID (group B, 3 horses). CSE, at the different dosages tested, was integrated into the feed twice a day, and administered for 59 days. The reduced size of group B, compared to that of group A and controls, was determined by the amount of the CSE available.

### 2.3. Clinical Evaluation and Scoring System

Each horse was subjected, to visits (Table S2, form of the clinical examination) composed of a ’general objective examination’ (GOE), and a ’particular objective examination of the locomotor apparatus’ (POELA). The parameters assessed for GOE were: The sensory state, heart rate, respiration rate, intestinal motility, mucosal status and capillary filling time. The parameters evaluated with the POELA were: palpation of the feet with a clamp; passive flexions of the neck (lateral, in extension and in ventro-flexion); passive flexions of the limbs (evaluation of their amplitude and sensitivity); evaluation of the animal in movement (step in a straight line, trot in a straight line, trot in left and right hand) and a global flexion test (hyperflexion of the limbs for 1 min, then trot for at least 20 m in a straight line); taking into account that the evaluation of the score of the flexion test is markedly influenced by the amount of force and technique applied, the performer of the test was the same for all the examinations. Since the CSE was supposed to ameliorate the severity of the symptoms and the fluency of the movements, rather than remove the causes of the osteoarthritic condition, imaging techniques for the diagnosis of the specific OA pathologies affecting the different subjects, were not considered. Some horses of all groups (included the highest dosage) with initial score values of 0 for several parameters of POELA were also included in this study, aiming to verify if spontaneously, or as a consequence of the CSE treatment, OA symptoms and/or pain perception might have occurred.

The animals, by choice, were examined ’cold’, in order to avoid possible false negatives in the evaluation of the different parameters, especially during the flexural tests. Each horse was always visited by the same veterinary practitioner who, however, didn’t know to which group the horses belonged. The scores of the POELA parameters, with the horse in standing position, were evaluated based on the presence (+) or absence (NN) of sensitivity during the manipulations; the more positive signs were detected, the higher was the degree of sensitivity attributed. To indicate reductions in the amplitudes of the articular angles during the manipulations, the scores were expressed as negative signs (−); in the case of a correctly maintained angle, the wording NN was used. To perform the statistical analysis, each score needed to be expressed with a numerical value; for this reason, according to the different parameters, the score value of 1 was attributed at each (+) or (−) sign; the formula NN, instead, corresponded to a score value of 0. In the case of the remaining parameters of POELA, that were assessed with the horse in movement, the veterinarians evaluated the single limbs by following the numerical 6-point scale (0–5) adopted by the American Association of Equine Practitioners (AAEP) for the assessment of lameness [15]. The total score of each horse for any parameter, was, therefore, obtained by adding the partial values of the scores related to each assessed limb. The study, which was carried out simultaneously with three groups, was realized from February to June. The horses of the control group and those of group A were subjected to GOE and POELA two times: At the beginning of the treatment (T0) and after 59 days (T59), this being the time that is given to detect amelioration in the AO symptomatology in the human species. In the hypothesis that the higher dosage would have resulted in undesired side effects of the CSE, the horses of group B were monitored more frequently, approximately every two weeks from T0 to T59 and one more time after 69 days after the end of the treatment (T128).

### 2.4. Laboratory Analysis and Questionnaire

At T0 and T59, blood samples from all the horses were taken from the jugular vein. Plasma and serum were than subjected to the following determinations, aimed to monitor both the general conditions of the animals (presence of infectious diseases, insurgence of novel inflammatory status, modifications of redox status etc.), and the appearance of side effects, due to the administration of the CSE similar to those described for NSAIDs (i.e., gastrointestinal protein loss revealed as by hypoalbuminemia) [16,17]: Serum protein electrophoresis, fibrinogen levels, quantification of TNFα on serum by ELISA test [18,19,20], detection of d-ROMs test (dosage- Reactive Oxygen Metabolites) [21] on plasma, and by FRAS test (Ferric Reducing Ability of Serum) [22] on serum. Every two weeks starting from T0, follow-up questionnaires (Table S3 follow up questionnaire) were submitted by the horse trainers.

### 2.5. Statistical Analysis

Quantitative data were evaluated as means +/− standard deviation and qualitative as the median and interquartile range (25–75%). Comparison among groups (control, groups A and B) was performed by means of Mann Whitney test or Kruskal–Wallis. Intra group comparisons were tested by means of Friedman and Wilcoxon test and ANOVA, as appropriate. All tests were two-sided with *p* < 0.05 considered statistically significant. All statistical analyses were performed with SPSS version 25 (IBM Statistics).

## 3. Results

The clinical parameters detected by GOE for the horses of all groups (control, A and B), that included some that should have revealed pain behavior, were within the physiological ranges at T0, and were not significantly modified during the entire time of the investigation (data not shown). The presence/maintenance of good general health conditions of the animals were further confirmed by the laboratory analysis on plasma and serum samples (data not shown). In particular, the unmodified electrophoretic profiles of the serum proteins suggest that no subject was affected by infectious diseases, and that in the case of the treated horses, the CSE didn’t cause protein loss (detected by hypoalbuminemia), due to lesions of the gastrointestinal tract, as it has been shown for high dosages and prolonged treatments with NSAIDs [16,17]. Moreover, the CSE didn’t modify either inflammatory or redox status of the treated subjects. A further indication of the safety of CSE came from the questionnaires collected through interviews with the trainers of the horses, that did not mention any undesired side effects, no alteration of their behavior and physiological functions. The owners and trainers also reported that the animals in group B moved with more elasticity and fluency.

**Figure 1:**
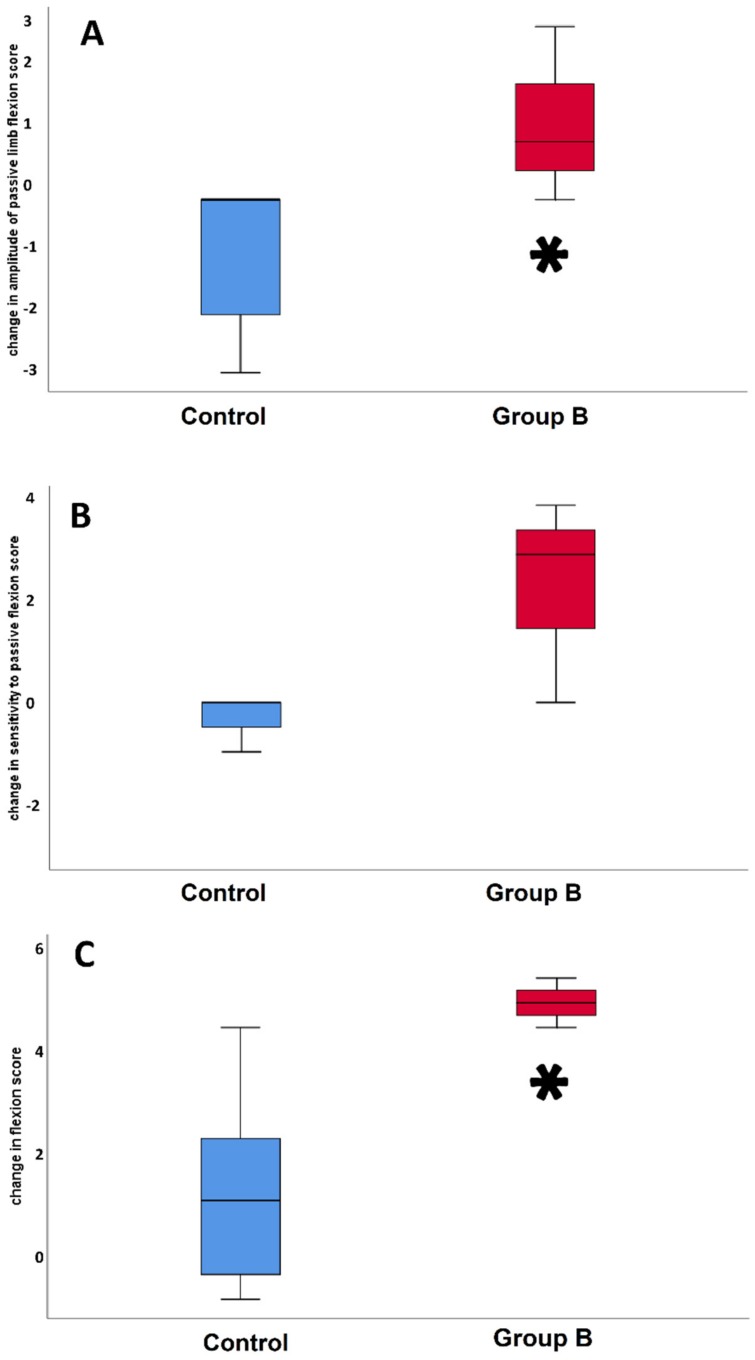
Box-plots of score change at time 0 and after 59 days for the parameters ’amplitude of passive flexion’ (**A**) (median of the Control group 0.0 with a range between −2.0 and 0.0, median of Group B 1.0 with a range between 0.5 and 2.0), ’sensitivity to passive flexion’ (**B**) (median of the Control group 0.0 with a range between −2.0 and 0.0, median of Group B 1.0 with a range between 0.5 and 2.0) and ’flexion test’ (**C**) of horses treated with 30 g BID CSE (Group B) (median of the Control group 1.0 with a range between −5.0 and 2.25, median of Group B 5.0 with a range between 4.75 and 5.25), and controls. The asterisk indicates *p* < 0.05.

With regard to POELA, only in the case of group B, the group that received the highest CSE dosage, 30 g BID, did the clinical results showed ameliorations compared to the control group for three parameters i.e.: (i) amplitude of passive limb flexion; (ii) sensitivity to passive limb flexion; and (iii) flexion test. The comparison of the score values for these parameters at T0 and T59 is reported in Figure 1.

In particular, statistical significance was observed for (i) and for (iii), with p values of 0.042 and 0.03, respectively. Interestingly also the comparison of the scores between the horses of groups A and B, showed improvements for the same parameters in the case of the animals treated with the 30 g BID dosage (not shown), that were significant for (i) and (iii), with *p* values of 0.033 and 0.017 respectively. These data clearly indicate that a dosage of CSE (7 g BID) comparable to that prescribed for humans, is not effective to induce any ameliorations of the symptoms of horses affected by chronic OA.

**Figure 2:**
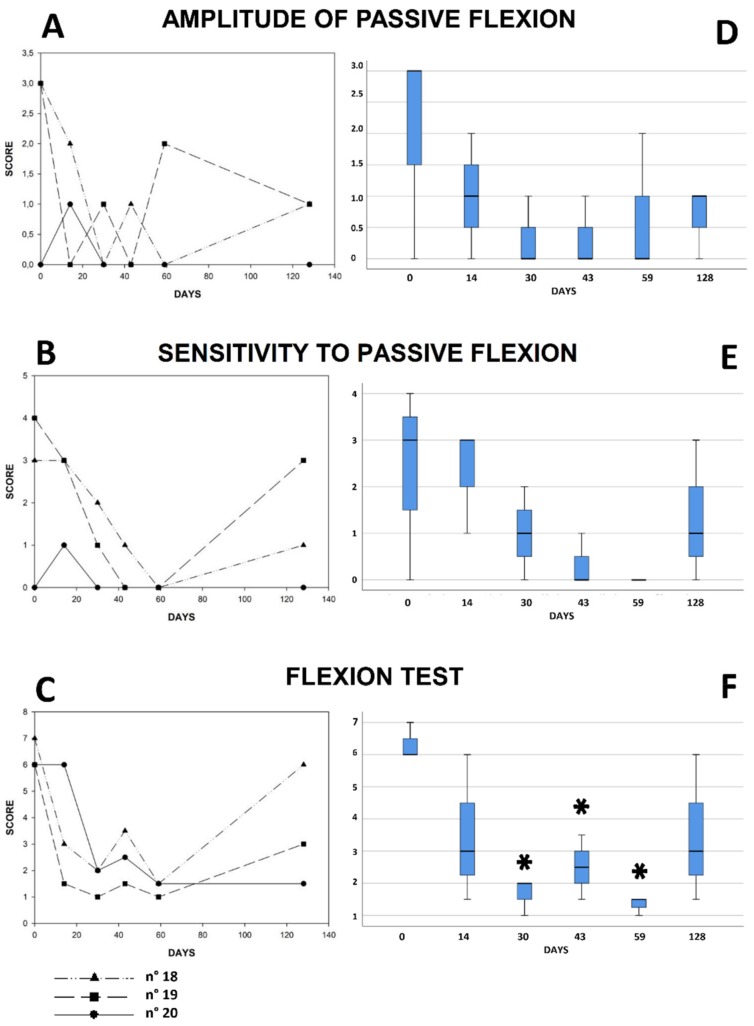
Graphs (**A**–**C**): Time profiles of the scores of the individual horses treated with CSE at the 30 g BID dosage monitored between T0 and T128, for the parameters ‘amplitude of passive flexion’, ‘sensitivity to passive flexion’, and ‘flexion test’. Graphs (**D**–**F**): Box-plots of the scores of the same horses (D: Chi square 3.91, *p* = 0.42. E: Chi square 9.03, *p* = 0.059. F: Chi square 11.08, *p* = 0.026). The asterisk indicates *p* < 0.05.

The graphs shown in Figure 2, which are based on score values from six different POELA evaluations of each horse of group B, at approximately two week intervals from T0 to T59, allow establishing both a clinical assessment of the scores of the parameters (i), (ii) and (iii) of the individual horses of group B as a function of time (Figure 2A–C), and a statistical evaluation of the variations of their median values with respect to the initial clinical conditions (Figure 2D–F).

The median scores of parameter (i), starting from day 14, remained constantly lower than that of day 0 until the end of the study (Figure 2A). For the two treated subjects (Nos 18 and 19) that showed higher score values at T0 (score value 3), the improvement observed after 14 days, was maintained (score values between 0 and 1) until day 49. At day 59, which corresponds to the interruption of the CSE administration, subject n° 18 showed a complete remission of the symptoms, while subject n° 19 showed an increase (score 2); after 69 days (T128) from the suspension of the treatment, both subjects showed a score value of 1. During the treatment, the scores of the horse that at the first visit did not show any symptoms regarding this parameter, except for a temporary increase (value of 1) after 14 days, maintained the score value of 0 for all the duration of the treatment. Although the experimental data indicate an amelioration of the symptomatology related to the parameter (i) for two of the treated horses, the high variability of the initial scores (values between 0 and 3) and the limited number of subjects examined, did not allow establishing statistical significance of the clinical data assessed after the first clinical examination (Figure 2D). Nevertheless, Figure 1 shows that while for the controls there was a worsening of the symptoms of parameter (i) between T0 and T59, for those of group C there was a statistically significant improvement of the values of the scores. Also, for parameter (ii), even if there was not a statistical significance of the data, the horses of group B ameliorated their condition starting from day 31, until the disappearance of the symptoms at day 59. Interestingly, 69 days after the interruption of the CSE treatment, the medians of the scores started to increase (Figure 2B,E), a trend that was particularly evident for the two horses (Nos. 18 and 19) with the highest initial values (3 and 4, respectively). The other treated subject (No 20), whose initial score was 0, remained stable, with a minimal oscillation at day 14 (value of 1), even after the suspension of the CSE treatment. In general, these data may indicate that CSE is effective in reducing the sensitivity to passive flexion, particularly in the two subjects showing more evident symptoms at T0. Regarding the parameter (iii), the treated horses showed a statistically significant reduction of the scores (*p* < 0.05) starting from day 31 (Figure 2C,F) along all the duration of the treatment; after its suspension, the same horses that exhibited a worsening of their score for (ii), also showed a worsening of their scores for (iii).

## 4. Discussion

In horses affected by chronic OA, the main goal of a symptomatic therapy, is the reduction of the pain aimed to improve the quality of life of the patient; moreover, although symptomatic therapies rarely contribute to an optimal recovery of the functionality of the limbs, they are essential to prevent the degeneration of the clinical condition of the horse [23]. These therapeutic protocols are generally based on NSAIDs that are not suitable for long-term treatments, because they cause undesirable side effects (e.g., gastritis). Therefore, in recent years, alternative therapeutic approaches to NSAIDs, that might improve the patient’s clinical condition over time, or at least stabilize it [24,25], are more frequently taken into consideration. CSE, whose anti-inflammatory properties have been preliminarily tested in laboratory animals, is prescribed, in the form of dietary supplement, for the treatment of chronic arthritis and osteoporotic diseases in humans. The active compounds contained in CSE that are thought to behave as competitive inhibitors for COX (cyclooxygenase) and LOX (lipoxygenase) [26,27], are supposed to produce analgesic and anti-inflammatory activities by inhibiting, through the same molecular mechanisms of NSAIDs (for this reason CSE is also called crypto-NSAID), the synthesis of leukotrienes and prostaglandins [26,27]. With regard to the effects of CSE on the symptoms of horses affected by chronic OA, the present study has shown that, only at the highest dosage tested of 30 g BID, do the score values of both sensitivity and amplitude of passive limb flexion, and the score values of the global flexion test, exhibit progressive, positive variations during the 59 days of product administration. In particular, the sensitivity to passive flexion decreased for all the horses from T0 to T59, and in the case of the subjects with the highest initial score values (Nos 18 and 19), it started to increase after the suspension of the CSE treatment (Figure 2B). Sensitivity to passive flexion, whose evaluation is determined in response to the mobilization of the main joints (carpus/tarsus, interphalangeal) of the limb imposed by the veterinarian, is an indicator of the degree of pain, due to osteoarticular damage; therefore, even in the absence of a statistical significance (Figure 2E), since the sensitivity of the passive flexion improves during the administration of the product in the two horses with the highest initial scores, it is plausible to suppose that CSE performs an analgesic action by reducing the pain response to limb flexion. The time profiles of the scores of the amplitude to passive flexion (Figure 2A), although not statistically significant (Figure 2D), reveal an improvement of the values in the two subjects exhibiting the most evident symptomatology at T0. This evidence could support the analgesic efficacy of CSE that had been hypothesized from the data of the sensitivity of the passive flexion. As further support to this hypothesis, the statistical relevance of the improvement in the clinical symptomatology obtained from the evaluation of the score values of the global flexion tests (Figure 2C,F) must be considered. This parameter, in general, evaluates the severity of the lameness (even in the asymptomatic form) as a function of time. In particular, if a joint affected by a chronic pathology (regardless of the etiology) is subjected to a flexion test, during the movement phase, there is a worsening of the limping of the subject, which is more severe as the symptom correlated to pain, lasts longer. In particular, the statistical analysis shows a significant improvement for all the CSE treated horses (Figure 2F), with p value lower than 0.05, already starting, in two subjects, from, T31. Flexion test is a fundamental parameter for the assessment of the clinical condition of the joint, including the connected anatomical structures (ligaments and tendons) which are subjected to mechanical stress, because it is positive even in the absence of a manifest lameness. In fact, chronic arthropathies frequently do not show serious or evident continuous signs of limping; therefore, a well-executed flexion test in some cases can detect even silent joint problems. Considering the significance of the statistical analysis of the time profiles of the scores of the flexion test in comparison with T0, in combination with the clinical improvement for the sensitivity and amplitude of passive flexions, it is plausible to attribute to CSE an analgesic effect that remains constant over time, while the product is administered to the horse. The reduction of the pain response, which allows the horses to improve joint mobility, has also been confirmed by the trainers who indicated in the surveys, that the 30 g BID CSE treated animals exhibit more elastic movements, are more dynamic and tolerate prolonged physical exercise in comparison with T0.

The reasons why in the horse the best responses occurred with a dosage (30 g BID), that is about 10 times higher than that recommended for humans remains to be defined; nevertheless, it can be hypothesized that it might reflect the different diets and food process mechanisms that characterize the two species. The horse, being a herbivore, has a gut microbiome that is more oriented to the chemical transformation and metabolism of compounds of vegetal origin than that of humans, which are omnivorous. On this basis, it could be speculated that the different metabolic elaboration capacity of food components could determine, in the horse, a more quantitative and/or faster inactivation of the components that are supposed to be responsible for the anti-inflammatory properties of CSE. As previously observed both in experimental animals and humans, and confirmed here in the horse, a relevant advantage of CSE in comparison to conventional anti-inflammatory drugs, is that it can be prescribed for protracted treatments, without unwanted side effects. In fact, both laboratory and clinical data form the present investigation showed that the health conditions of the animals didn’t change over a 59 days treatment. The most dangerous side effects of NSAIDs is the induction of ulcers in the gastrointestinal tract that, in the case of high dosages and prolonged treatments, can be detected by hypoalbuminemia and clinically evident edematous conditions, that were not found in the horses treated with CSE. The lack of side effects of a product like CSE, whose anti-inflammatory/analgesic properties are considered to be the same that characterize NSAIDs, certainly represents an intriguing issue. The answer to this question, could probably reside in the different delivery, turnover, and inhibitory activities for COX and LOX of the bioactive components of the CSE and NSAIDs in-vivo. Since the anti-inflammatory activity of CSE is probably determined by a mixture of bioactive compounds, their relative concentrations, synergies or mutual inhibition should be probably considered to understand the differences with NSAIDs. A possible answer to the lack of gastrolesivity by CSE, as mentioned above [14], could be due to its gastroprotective effect, mediated through non-prostaglandin mechanisms.

Moreover, the lack of interference on cytochrome P450 activity reported in the literature for the murine and human species [28], would represent a further index on the safety of the use of CSE also in equine medicine. In conclusion, although preliminary, the results presented here have shown on one side the analgesic properties of CSE in horses affected by OA, as well its possible application for prolonged treatments. Nevertheless it must be underlined that a larger number of treated subjects, as well as other variables which here have not been considered, like clinical severity, age, type of nutrition, attitude, type of pathology that gave rise to the OA status, the use of instrumental diagnostic tools for a more accurate monitoring of the clinical conditions of the animals, will be certainly necessary to better define protocols for its effective applications in equine orthopedics.

## Supplementary Materials

The following are available online at https://www.mdpi.com/2076-2615/9/8/585/s1, Table S1 characteristics of the subjects enrolled in the trial and experimental data collected; Table S2 form of the clinical examination; Table S3 follow up questionnaire.

## References

[1] Caron J.P., Genovese R.L., Ross M.W., Dyson S.J. (2008). Principles and practices of joint disease treatment. Diagnosis and Management of Lameness in the Horse.

[2] Goldring S.R., Goldring M.B. (2006). Clinical aspects, pathology and pathophysiology of osteoarthritis. J. Musculoskelet. Neuronal Interact..

[3] Curtiss J., Paul H. (1973). The pathophysiology of joint infections. Clin. Orthop. Relat. Res..

[4] Brandt K.D., Dieppe P., Radin E.L. (2009). Etiopathogenesis of osteoarthritis. Med. Clin. N. Am..

[5] Goodrich L.R., Nixon A.J. (2006). Medical treatment of osteoarthritis in the horse: A review. Vet. J..

[6] Chevallier A. (1996). The Encyclopedia of Medicinal Plant.

[7] Gaby A.R. (1999). Natural treatments for osteoarthritis. Altern. Med. Rev..

[8] Trumble T.N. (2005). The use of nutraceuticals for osteoarthritis in horses. Vet. Clin. N. Am. Equine Pract..

[9] McIlwraith C.W., Frisbie D.D., Kawcak C.E. (2012). The horse as a model of naturally occurring osteoarthritis. Bone Joint Res..

[10] Powanda M.C., Rainsford K.D. (2011). A toxicological investigation of a celery seed extract having anti-inflammatory activity. Inflammopharmacology.

[11] Whitehouse M.W., Roberts M.S., Brooks P.M. (1999). Over the counter (OTC) oral remedies for arthritis and rheumatism: How effective are they?. Inflammopharmacology.

[12] Zhu L.H., Bao T.H., Deng Y., Li H., Chen L.X. (2017). Constituents from Apium graveolens and their anti-inflammatory effects. J. Asian Nat. Prod. Res..

[13] Sowbhagya H.B. (2014). Chemistry, Technology, and Nutraceutical Functions of Celery (Apium graveolens L.) An Overview. Crit. Rev. Food Sci. Nutr..

[14] Whitehouse M.W., Butters D.E., Clarcke M.L., Rainsford K.D. (2001). NSAID gastropathy: Prevention by celery seed extracts in disease-stressed rats. Inflammopharmacology.

[15] Kester W.O. (1991). Definition and classification of lameness. Guide for Veterinary Services and Judging of Equestrian Events.

[16] MacAllister C.G., Morgan S.J., Borne A.T., Pollet R.A. (1993). Comparison of adverse effects of phenylbutazone, flunixin meglumine, and ketoprofen in horses. J. Am. Vet. Med. Assoc..

[17] Noble G., Edwards S., Lievaart J., Pippia J., Boston R., Raidal S.L. (2012). Pharmacokinetics and safety of single and multiple oral doses of meloxicam in adult horses. J. Vet. Intern. Med..

[18] Tamzali Y., Guelfi J.F., Braun J.P. (2001). Plasma fibrinogen measurement in the horse: Comparison of Millar’s technique with a chronometric technique and the QBC-Vet Autoreader™. Res. Vet. Sci..

[19] Crisman M.V., Scarratt W.K., Zimmerman K.L. (2008). Blood proteins and inflammation in the horse. Vet. Clin. N. Am. Equine Pract..

[20] Borges A.S., Divers T.J., Stokol T., Mohammed O.H. (2007). Serum iron and plasma fibrinogen concentrations as indicators of systemic inflammatory diseases in horses. J. Vet. Intern. Med..

[21] Kusano K., Yamasaki M., Kiuchi M., Kaneko K., Koyama K. (2016). Reference range of blood biomarkers for oxidative stress in Thoroughbred racehorses (2–5 years old). J. Equine Sci..

[22] Benzie I.F.F., Strain J.J. (1996). The ferric reducing ability of plasma (FRAP) as a measure of “antioxidant power: The FRAP assay”. Anal. Biochem..

[23] Baxter G.M. (2011). Adams and Stashak’s Lameness in Horses.

[24] Blumenthal M., Busse W.R., Goldberg A., Gruenwald J., Hall T., Riggins C.W., Klein S., Rister R.S. (1998). The Complete German Commission E Monographs—Therapeutic Guide to Herbal Medicines.

[25] De Grauw J.C., Van De Lest C.H., Van Weeren R., Brommer H., Brama P.A. (2006). Arthrogenic lameness of the fetlock: Synovial fluid markers of inflammation and cartilage turnover in relation to clinical joint pain. Equine Vet. J..

[26] Beluche L.A., Bertone A.L., Anderson D.E., Rohde C. (2001). Effects of oral administration of phenylbutazone to horses on in vitro articular cartilage metabolism. Am. J. Vet. Res..

[27] Momin R.A., Nair M.G. (2002). Antioxidant, cyclooxygenase and topoisomerase inhibitory compounds from Apium graveolens Linn. Seeds. Phytomedicine.

[28] Mencherini T., Cau A., Bianco G., Della Loggia R., Aquino R.P., Autore G. (2007). An extract of Apium graveolens var. dulce leaves: Structure of the major constituent, apiin and its anti-inflammatory properties. J. Pharm. Pharmacol..

