# Supplementary material for: Clinical Effects of the Extract of the Seeds of the Indian Celery—Apium graveolens—In Horses Affected by Chronic Osteoarthritis
Source: Animals (Basel). 2019 Aug 20;9(8):585. doi: 10.3390/ani9080585 (PMC6720213; doi:10.3390/ani9080585)
Supplement: Supplementary file 1 [file animals-09-00585-s001.zip › S3 follow up form.pdf]

FOLLOW UP (Trainer, Owner)

**HORSE:**
**SEX:**
**ATTITUDE:**
**AGE (YEARS):**

The subject willingly takes the product:	YES	NO
The subject regularly takes the product:	YES	NO
There have been variations in physiological functions:	YES	NO
You have noticed some changes / attitudes in the subject since he took the supplement:	YES	NO
The animal is always alert and is not reluctant to move:	YES	NO
There are obvious signs of sweating:	YES	NO

The gait is regular:	YES	NO
The riding gait is regular:	YES	NO
You noted the appearance of areas of pain (neck, back, limbs):	YES	NO
NOTES:		
