# Supplementary material for: Clinical Effects of the Extract of the Seeds of the Indian Celery—Apium graveolens—In Horses Affected by Chronic Osteoarthritis
Source: Animals (Basel). 2019 Aug 20;9(8):585. doi: 10.3390/ani9080585 (PMC6720213; doi:10.3390/ani9080585)
Supplement: Supplementary file 1 [file animals-09-00585-s001.zip › S2 visit form.pdf]

CLINICAL SHEET FOR THE VETERINARY PRACTITIONERS VISITING THE HORSES PARTICIPATING IN THE CSE TRIAL

HORSE:			SEX	ATTITUDE
BREED	COAT COLOUR	AGE	DESIGNATION	N° MICROCHIP

GENERAL OBJECTIVE EXAMINATION (GOE)

**TEGUMENTARY EXAMINATION (scars, excoriations, wounds, swelling, etc ...)**
LEFT FORE LIMB
RIGHT FORE LIMB
LEFT HIND LIMB
RIGHT HIND LIMB
OTHER

**RESPIRATORY SYSTEM EXAMINATION**
note:

**CARDIOCIRCULATORY SYSTEM EVALUATION**
note:

PARTICULAR OBJECTIVE EXAMINATION OF THE LOCOMOTOR APPARATUS (POELA)

PERPENDICULARITY	LEFT FORE LIMB
	RIGHT FORE LIMB
	LEFT HIND LIMB
	RIGHT HIND LIMB

FEET PALPATION	LEFT FORE LIMB	SENSITIVITY (NO; +; ++; +++):	AREA
	RIGHT FORE LIMB	SENSITIVITY (NO; +; ++; +++):	AREA
	LEFT HIND LIMB	SENSITIVITY (NO; +; ++; +++):	AREA
	RIGHT HIND LIMB	SENSITIVITY (NO; +; ++; +++):	AREA

PASSIVE FLEXION	LEFT FORE LIMB	AMPLITUDE (OK; -; - -; - - -):	SENSITIVITY (NO; +; ++; +++):
	RIGHT FORE LIMB	AMPLITUDE (OK; -; - -; - - -):	SENSITIVITY (NO; +; ++; +++):
	LEFT HIND LIMB	AMPLITUDE (OK; -; - -; - - -):	SENSITIVITY (NO; +; ++; +++):
	RIGHT HIND LIMB	AMPLITUDE (OK; -; - -; - - -):	SENSITIVITY (NO; +; ++; +++):

EVALUATION OF GAIT						
	STRAIGHT WALK		LEFT FORE LIMB /5	RIGHT FORE LIMB /5	LEFT HIND LIMB /5	RIGHT HIND LIMB /5
	STRAIGHT TROT		LEFT FORE LIMB /5	RIGHT FORE LIMB /5	LEFT HIND LIMB /5	RIGHT HIND LIMB /5
	COUNTER TROT LEFT HAND		LEFT FORE LIMB /5	RIGHT FORE LIMB /5	LEFT HIND LIMB /5	RIGHT HIND LIMB /5
	COUNTER TROT RIGHT HAND		LEFT FORE LIMB /5	RIGHT FORE LIMB /5	LEFT HIND LIMB /5	RIGHT HIND LIMB /5
	FLEXION TEST		LEFT FORE LIMB /5	RIGHT FORE LIMB /5	LEFT HIND LIMB /5	RIGHT HIND LIMB /5
